# Causal models of rate-independent damping in insect exoskeleta

**DOI:** 10.1242/jeb.249940

**Published:** 2025-07-07

**Authors:** Arion Pons

**Affiliations:** Division of Fluid Dynamics, Department of Mechanics and Maritime Sciences, Chalmers University of Technology, 412 96 Gothenburg, Sweden

**Keywords:** Insect flight motor, Exoskeleton, Damping, Hysteretic, Rate-independent

## Abstract

In insect locomotion, the transmission of energy from muscles to motion is a process within which there are many sources of dissipation. One significant but understudied source is the structural damping within the insect exoskeleton itself: the thorax and limbs. Experimental evidence suggests that exoskeletal damping shows frequency (or rate) independence, but investigation into its nature and implications has been hampered by a lack methods for simulating the time-domain behaviour of this damping. Here, synergising and extending results across applied mathematics and seismic analysis, I provide these methods. Existing models of exoskeletal rate-independent damping are equivalent to an important singular integral in time: the Hilbert transform. However, these models are strongly noncausal, violating the directionality of time. I derive the unique causal analogue of these existing exoskeletal damping models, as well as an accessible approximation to them, as Hadamard finite-part integrals in time, and provide methods for simulating them. These methods are demonstrated on several current problems in insect biomechanics. Finally, I demonstrate, for the first time, that these rate-independent damping models show counterintuitive energetic properties – in certain cases, extending to violation of conservation of energy. This work resolves a key methodological impasse in the understanding of insect exoskeletal dynamics and offers new insights into the micro-structural origins of rate-independent damping as well as the directions required to resolve violations of causality and the conservation of energy in existing models.

## Teaser

**Summary:** Energy dissipation within the insect exoskeleton is an important factor within a range of locomotive behaviors. I provide new mechanical models of this dissipation phenomenon, and methods to simulate them.

## INTRODUCTION

Exoskeletal structures play crucial roles in insect locomotion. In winged insects (Pterygota), the wings themselves are exoskeletal outgrowths originating from ancestral structures of uncertain form (Engel et al., 2013), and the thoracic exoskeleton contains not only the mechanisms that translate the action of the flight muscles into wingbeat motion (Melis et al., 2024; Walker et al., 2014), but also (in certain cases) structures that can store elastic energy and thereby reduce wingbeat power requirements (Gau et al., 2019; Warfvinge et al., 2017). In walking, jumping and hopping insects across orders, the exoskeleton of the legs and thorax provides contact and support forces, and can contribute to energy storage for use in jumps and bounds (Bolmin et al., 2021; Burrows, 2010). Studies of the energetics of insect locomotion focus often on the elastic properties of these exoskeletal structures – it is, after all, these elastic properties that enable improvements in flight efficiency (Gau et al., 2019; Pons et al., 2023) and jumping performance (Bolmin et al., 2021; Burrows, 2010). But damping is also at work: exoskeletal structures also dissipate energy via viscoelastic effects within the partially sclerotised chitin–protein matrix of which they are composed (Aberle et al., 2017). Empirical evidence from dynamic mechanical analysis (DMA) across a range of species and locomotor systems – including hawkmoth flight motors (Gau et al., 2019; Wold et al., 2023), beetle elytra (Lomakin et al., 2010) and cockroach legs (Dudek and Full, 2006, 2007) – indicates that this damping is often largely frequency- or rate-independent, a property consistent with the characteristics of chitin and other cuticle polymers (Aberle et al., 2017; Jensen and Weis-Fogh, 1962; Sun et al., 2016), and that can lead to favourable control properties (Dudek and Full, 2007; Wold et al., 2023).

Linear rate-independent damping – otherwise termed structural or frequency-independent damping – permits a straightforward and convenient model representation in the frequency domain: a constant imaginary value appended to the stiffness term within the force–displacement transfer function, often styled *i*γ (Dudek and Full, 2006; Gau et al., 2019; Wold et al., 2023). However, this straightforwardness conceals serious practical and analytical problems. Exact time-domain formulations of rate-independent damping are absent from biomechanical literature, and indeed, one finds this coefficient in no table of inverse Fourier transforms. Without such formulations, there is no accurate way to identify the classical damping parameter γ from time-domain exoskeletal mechanical data, particularly when nonlinear effects are present, and frequency-domain analysis breaks down (Lynch et al., 2021; Pons and Beatus, 2022a). Neither is there any way to simulate time-domain models of the exoskeleton, e.g. as part of a full bio-aero-structural model of an insect flight motor (Gau et al., 2023; Pons, 2023). However, parallel studies in the field of seismic analysis (Inaudi and Makris, 1996; Luo and Ikago, 2021) have made progress on time-domain modelling of same classes of damping model, and offer pathways for overcoming these biomechanical challenges. Despite the wide difference in field, vibrational deformation of built structures within earthquakes resembles that of exoskeletal structures during insect locomotion in more ways than one: in cantilever vibration in running (Dudek and Full, 2007), in complex modeshapes in the flight motor (Ando and Kanzaki, 2016) and in properties of terrestrial soil, which itself shows rate-independent damping (Kausel and Assimaki, 2002).

In this work, I address these issues by developing time-domain formulations of linear rate-independent damping and applying them to problems in the identification of insect exoskeletal damping, a process based on methods across applied mathematics, seismic analysis and biomechanics. First, leveraging techniques from seismic analysis (Inaudi and Makris, 1996; Makris, 2017), I present an exact time-domain formulation for rate-independent damping based on the singular integral known as the Hilbert transform. In doing so, I confirm this damping to be non-causal, that is, in violation of the directionality of time. Following and extending further existing results (Makris, 1997a; Pons, 2024), I resolve the causality violation and obtain a pair of approximate causal time-domain models, supported by new and accessible numerical methods for computing the singular integrals associated with these models. Next, I apply these causal and non-causal models to challenging problems in entomological literature, including the identification of rate-independent damping parameters from exoskeletal transient responses and DMA data, accounting for nonlinear stiffness and motion asymmetry (Dudek and Full, 2007; Wold et al., 2023), and time-domain simulation of structural models with rate-independent damping (Dudek and Full, 2007; Gau et al., 2023). Via these simulations, I reveal a crucial and previously unknown caveat of linear rate-independent damping: such damping is not always dissipative, that is, it is capable of releasing energy, as well as absorbing it. In doing so, I identify new restrictions on the use and validity of these damping models. Finally, I identify pathways toward more clearly understanding the mechanistic origins of and best-practice models for rate-independent damping. Synergising applied mathematics, seismic analysis and insect biomechanics, these results provide wider integrative studies of insect locomotion with tools to integrate rate-independent damping within time-domain models of insect locomotion processes, as well as a more detailed understanding of the limits of validity of such models and the ways in which they can break down.

## FORMULATIONS OF RATE-INDEPENDENT DAMPING

### Motivation and frequency-domain formulation

**Fig. 1.**
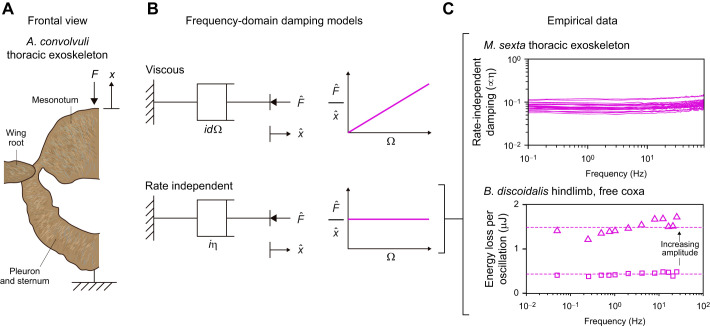
**Context for rate-independent exoskeletal damping.** (A) Frontal view of the thoracic exoskeleton of the hawkmoth *Agrius convolvuli*, after Ando et al. (2022), illustrating a dynamic mechanical analysis (DMA) context for structural damping within hawkmoth thoraces during flight (Wold et al., 2023). (B) Viscous and rate-independent models of thoracic damping, with associated scaling of force with frequency (Ω). (C) Reported DMA data for exoskeletal structures indicate rate-independent scaling: the thorax of the hawkmoth *Manduca sexta* (Gau et al., 2019); and the hindlimb of the false death’s head cockroach, *Blaberus discoidalis* (Dudek and Full, 2006).

The challenges that arise in existing models of rate-independent damping in the insect exoskeleton are given context by the unique circumstances in which these models have been constructed. Consider a classical single degree-of-freedom (1DOF) viscous damper, for instance, as might initially be taken to model the structural damping associated with thoracic deformation in dipteran and hymenopteran flight (Fig. 1A) (Lynch et al., 2021; Wold et al., 2023). This damper can be expressed in the time (*t*∈ℝ) and frequency (Ω∈ℝ) domains as:
(1)

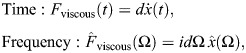

where *d* is the viscous damping parameter and *F* is the action force (the force required to displace the damper at velocity 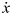). Note that the reaction force is –*F*, and that the relationships between time and frequency domains are given by the Fourier and inverse Fourier transforms. For generic function *G*(*t*):
(2)

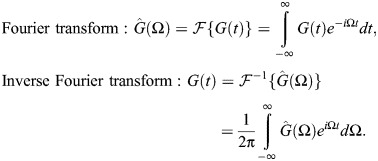


In Eqn 1, the damping force is frequency dependent: 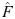
scales by Ω (Fig. 1B). Empirical data for a range of insect exoskeletal structures contradict this viscous model (Dudek and Full, 2006, 2007; Gau et al., 2019; Lomakin et al., 2010; Wold et al., 2023), and indicate rather that this force amplitude (and associated metrics, such as the energy loss per oscillation) is largely independent of frequency, over several orders of magnitude. Several such data are illustrated in Fig. 1C.

To capture rate-independent (RI) damping, common practice (Dudek and Full, 2006; Lynch et al., 2021; Pons, 2023) is to make an ad hoc modification to Eqn 1, deleting the factor of Ω:
(3)

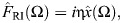
and defining a new rate-independent damping parameter η (Fig. 1B). However, as a matter of formal correctness, for Eqn 3 to be real-valued in the time domain, it must be defined as odd-symmetric in Ω, as (Luo and Ikago, 2021; Reggio and De Angelis, 2015):
(4)

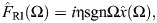
where sgn· is the signum function. In exoskeletal analysis, this damping model is typically combined with a linear or nonlinear stiffness to form a Voight-type viscoelastic model (Dudek and Full, 2007; Wold et al., 2023). In this context, we can also define the damper's viscoelastic storage (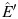) and loss (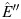) moduli, together defining its complex modulus (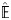):
(5)

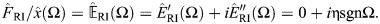


As can be seen, the rate-independent damper itself has zero storage modulus, but this will not be the case for all rate-independent dampers, as shown in the next three subsections.

### The ideal rate-independent damper

The exact time-domain formulation of the rate-independent model, Eqns 4 and 5, is known in seismic analysis literature (Inaudi and Kelly, 1995; Keivan et al., 2018). It is:
(6)

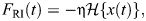
where 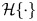 is the Hilbert transform, a real-valued singular integral transform representing the principal value (p.v.) convolution (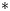) of the input motion with the kernel 1/π*t* (Poularikas, 2018):
(7)

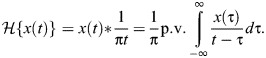


For illustration, Fig. 1 compares the response of a rate-independent damper (Eqn 7) with that of a viscous damper (Eqn 1), for a triangle wave input. Practically, evaluating the Hilbert transform is straightforward. A wide range of analytical results have been tabulated (King, 2009; Poularikas, 2018), and numerical implementations, in the form of the discrete Hilbert transform (DHT), are available in many scientific computing languages: in MATLAB, as hilbert(); in Python, as hilbert() within SciPy; and in R, via hilbert() within the gsignal package or HilbertTransform() within the hht package. An implementation in MATLAB is available within Dataset 1.

**Fig. 2.**
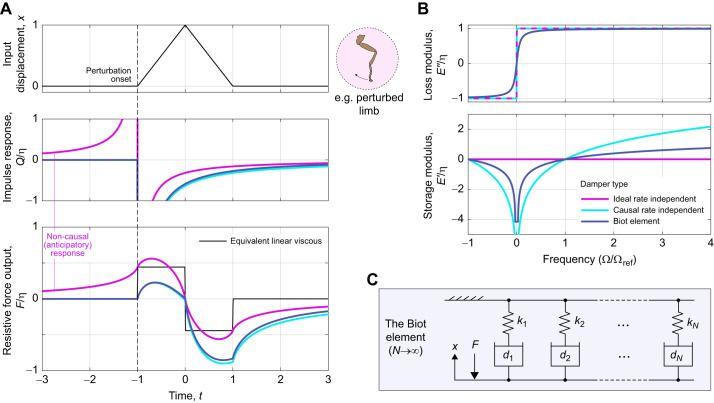
**Behaviour of rate-independent dampers: ideal, causal and the Biot element.** (A) Time-domain damping responses to a triangle pulse, illustrative of a transient perturbation to an insect limb. Damper impulse responses (*Q*/η, about perturbation onset) and force responses (*F*/η) are shown, computed via the numerical methods described in the Appendix, and for tuning parameter values Ω_ref_=π/2 and *N*_ref_=20. In these, the non-causal behaviour of the ideal rate-independent damper can be clearly seen. A linear damper of equivalent net power dissipation is included for comparison. (B) Loss and storage moduli of these dampers. Together, causality and loss modulus rate-independence strictly necessitate a logarithmic storage modulus. (C) Notably, the Biot element can be constructed as the infinite sum of linear stiffnesses and viscous dampers, illustrating a physical mechanism via which rate-independent damping can arise.

However, evaluating the Hilbert transform comes with a more fundamental limitation: as per Eqn 6, an integral must be performed over all time, both past and future. As such, the ideal rate-independent damper (Eqns 4 and 5) is non-causal: it does not respect the directionality of time (Makris, 1997b), but instead anticipates future events (Fig. 2A). One visualisation of the strength of this anticipatory response is the damper's impulse response, or memory function (Enelund and Olsson, 1999; Makris, 1997b), which is:
(8)

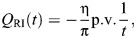
where p.v.1/*t* denotes a regularisation of the ordinary function 1/*t* (Kanwal, 1997; King, 2009). This impulse response is illustrated in Fig. 2A. It indicates that this damper is strongly non-causal: it weighs information from the past and future exactly equally. This property is an obstacle to time-domain simulation of exoskeletal structures, such as flight motor models (Gau et al., 2023; Lynch et al., 2021), and by its nature cannot exactly represent any real physical structure. These concerns motivate a search to find alternative models of rate-independent damping.

### The causal rate-independent damper

Querying methods by which the non-causal model of Eqns 4 and 5 could be turned into an equivalent causal model leads to a fundamental feature of viscoelastic theory. The causality of a viscoelastic structure is determined by the relationship between its storage (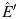) and loss (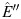) moduli, according to Titchmarsh's theorem and its generalisations (Nussenzveig, 1972; Pons, 2024). Practically, to turn the ideal rate-independent damper into a causal rate-independent (CRI) damper, a specific storage modulus must be added (Makris, 1997a; Pons, 2024):
(9)

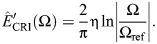
Ω_ref_>0 is a free parameter of the model: the reference, or threshold frequency, which is equivalent to a linear stiffness parameter (see Supplementary Materials and Methods). The complete complex modulus of the causal rate-independent damper is thus:
(10)

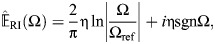
which is illustrated in Fig. 2 (cf. Eqn 5). Extending results from seismic analysis (Makris, 1997a), as per the Supplementary Materials and Methods, allows the derivation of time-domain formulations of this causal damper. Its impulse response is:
(11)

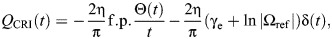
and its general force response is:
(12)

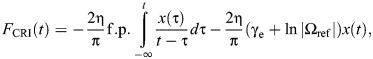
where γ_e_≈0.577 is the Euler–Mascheroni constant. Both results are illustrated in Fig. 2. In Eqns 11 and 12, f.p. denotes Hadamard finite-part integration, a technique for extracting finite values from diverging (infinite-valued) integrals.

Evaluating Eqn 12 for general exoskeletal input data is challenging. A description and validation of novel numerical methods for doing so is presented in the Appendix. A derivation in Supplementary Materials and Methods 2 and an implementation in MATLAB within Dataset 1. These numerical methods enable the simulation illustrated in Fig. 2, which confirms that this rate-independent damping model is indeed causal. However, this causality has come at a cost. The damper now has a storage modulus (Eqn 9), which is not only contrary to the intent of a damping model, but is also negative-valued at frequencies below Ω_ref_, and tends toward negative infinity in the quasistatic limit (Ω→0). As discussed in more detail later (see ‘Energetic properties of rate-independent damping’), Ω_ref_ can be set to ensure that any specified exoskeletal motion, *x*(*t*), will only elicit positive storage modulus. However, no Ω_ref_ can completely eliminate the trend to infinite negative storage modulus, and this raises a pair of new concerns. First, that infinite negative storage modulus is non-physical, and so this damper also can only be an idealisation (limit case) of a real physical structure. Second, that if the exoskeletal motion is not known in advance – e.g. in a simulation of an exoskeletal structure (see Gau et al., 2023) – then it is not possible to guarantee positive storage modulus. These concerns motivate a final alteration to this rate-independent damping model.

### The Biot element

The causality analysis used to derive Eqn 10 (Pons, 2024) implies that no other causal models show perfectly rate-independent loss modulus. The only pathway toward alleviating the infinite negative storage modulus of Eqn 10 involves approximating rate independence with some form of limited rate dependence. Several such approximations are available in structural dynamics and seismic analysis literature: from convenient but inaccurate approximations valid over only a narrow frequency band (Liu and Ikago, 2022; Reggio and De Angelis, 2015), to more complex generalised models that approach true rate independence in some parameter limit (Caughey, 1962; Luo and Ikago, 2021). Exoskeletal DMA data provide evidence for rate independence over a very large frequency range – up to three orders of magnitude (Dudek and Full, 2006; Gau et al., 2019) – and so we require a strong model. Here, I select the Biot element (Biot, 1958; Caughey, 1962; Luo and Ikago, 2021), with loss and storage moduli:
(13)

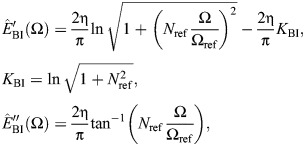
as illustrated in Fig. 2B, and where *K*_BI_ is defined for notational utility. This model has two free parameters: a reference frequency (Ω_ref_) and a shaping factor (*N*_ref_). Ω_ref_ specifies the transition from positive to negative storage modulus, and *N*_ref_ specifies how closely the Biot element approximates the causal rate-independent damper; e.g. for large *N*_ref_, the loss modulus approaches rate independence, but the quasistatic storage modulus approaches negative infinity. I suggest *N*_ref_≈20 for practical applications, as illustrated in Fig. 2B. Then at any given Ω_ref_, the loss modulus is 96% of its asymptote, but the storage modulus drops to a minimum of only –4.2η.

The Biot element is causal. This may be seen in its impulse response, derived in Supplementary Materials and Methods 1, and which to the author's knowledge is presented for the first time here:
(14)

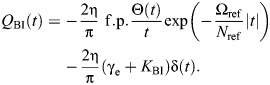


The Biot element's general force response is thus:
(15)

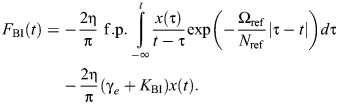
Evaluating Eqn 15 numerically is again challenging, but numerical methods developed for the causal rate-independent damper can be adapted: a description and validation of these methods is found in the Appendix, a derivation in Supplementary Materials and Methods 2, and an implementation in MATLAB is available within Dataset 1.

Finally, the Biot element is also physically realisable, as an infinite series of simple linear springs and viscous dampers, arranged in a generalized Maxwell configuration (Fig. 2C). More specifically, the Biot element arises in the case in which individual stiffnesses (*k_i_*) are inversely proportional to relaxation times (*r_i_*=*k_i_*/*d_i_*), *k_i_*
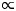
1/*r_i_*, implying that individual damping coefficients show a slowly increasing trend with stiffness, 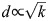. Details of this derivation are given in Caughey (1962) and Luo and Ikago (2021). In addition, based on the principle that the Biot element approaches the causal rate-independent damper as *N*_ref_→∞, the latter should also be regarded as physically realisable (at least, in the limit). The generalized Maxwell construction of these models not only confirms the realisable nature of this damping, but also offers insight into one mechanism by which rate-independent damping can arise in an exoskeleton: as a bulk effect caused by a statistical distribution of elastic and viscous mesoscale or microscale structures. I discuss this, and other mechanisms, in the Discussion.

### Equivalences

Above, I presented a series of three rate-independent damping models, defined in the time domain, and each addressing particular obstacles raised by the previous (causality, and infinite negative storage modulus). The end result is the Biot element, which suffers from none of the pathologies of the ideal rate-independent damper, but is considerably more complex, and comes with a storage modulus and a range of tuning parameters. However, for sinusoidal motion at any frequency Ω*, all three damping models can be made equivalent: the storage modulus of the causal damper and Biot element can be set to zero by setting Ω_ref_=Ω*, and the Biot element will show only slightly smaller loss modulus (according to *N*_ref_). The ideal damper is still non-causal, but in this circumstance, it behaves no different to its causal analogues. As such, for motion that is close to sinusoidal, the ideal rate-independent damper likely shows sufficient realism that it can be used as a convenient approximation. It is in the case of strongly non-sinusoidal motion that the other two damping models become relevant. Both in realism, and in a slightly more convenient evaluation, the Biot element is superior to the causal rate-independent damper: the former is merely the latter with an extra parameter (*N*_ref_), and the latter is a limit case of the former with a specific connection to the original ideal rate-independent damper.

## APPLICATION TO EXOSKELETAL MODELLING

### Time-domain identification based on exoskeletal DMA data

Using the time-domain models described above, progress can be made on several challenging problems in insect biomechanics that pertain to rate-independent damping. The first challenge was raised by Gau et al. (2019) and Wold et al. (2023) in the context of rate-independent damping within the exoskeleton of hawkmoths (*Manduca sexta*) within flight. The challenge is that existing frequency-domain methods for identifying rate-independent damping are restricted to a very specific form of data: loss moduli, measurable when a structure that behaves linearly is subjected to sinusoidal excitation during DMA. If loss moduli cannot be estimated owing to nonlinear effects – e.g. nonlinear elasticities, as per Gau et al. (2019) – then frequency-domain identification breaks down. And if we wish to study the case of non-sinusoidal excitation, as per Wold et al. (2023), to better understand thoracic damping effects during insect flight manoeuvres, then frequency-domain identification again breaks down, and worse, we have no method of confirming whether a rate-independent damping model remains valid.

**Fig. 3.**
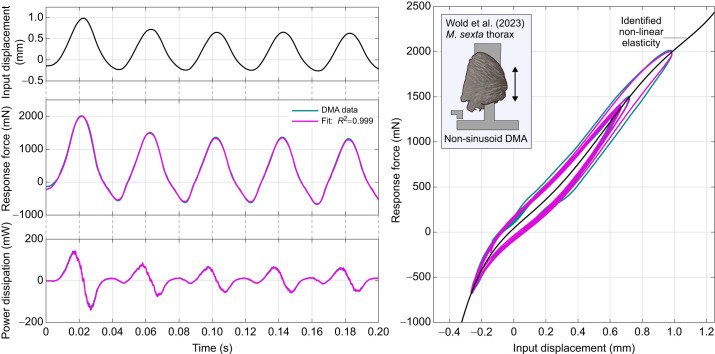
**Time-domain identification of rate-independent damping in the asymmetric DMA data of** **Wold et al. (2023)****, for a *Manduca sexta* thorax, accounting for nonlinear elasticity up to fifth-order polynomial (Eqn 16)**. Illustrated is a sample interval, 0≤*t*≤0.2 of the DMA input displacement signal, the response force data and fitted profile, and the response and fit power dissipation, as well a response and fit work loop over the complete fit interval, 0≤*t*≤1. Note that the displacement and force data are subjected to Savitsky–Golay smoothing (fifth order over a frame width of 5.1 ms) in order to eliminate high-frequency non-dissipative noise.

These challenges are solved by the time-domain formulations described above. In Fig. 3, I demonstrate fitting a rate-independent damper with a nonlinear elastic term to DMA data for a hawkmoth exoskeleton under non-sinusoidal ‘asymmetric’ excitation (Wold et al., 2023). The fitted model is:
(16)

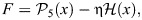

for force *F* (mN) and displacement *x* (mm). This time-domain model represents an ideal rate-independent damper, –η
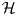
(*x*), in parallel with a nonlinear elasticity defined by a fifth-order polynomial in *x*, 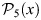, i.e.:
(17)

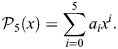
The fit parameters are η and *a*_0_–*a*_5_. Fitting is performed in the time-domain using a least-squares method; Fig. 3 illustrates the fitted result in time and in a work loop. An accurate fit (*R*^2^=0.999) results, indicating that a rate-independent damping is suitable, at least for this individual time series. The estimate of η is 277 N m^−1^, and if a dimensionless damping term γ is defined as η=a_1_γ, then this corresponds to γ=0.167, appropriate for the hawkmoth thorax (Wold et al., 2023). This is an illustrative solution to the challenge posed by Wold et al. (2023): it allows us not only to accurately study the case of non-sinusoidal input signal, but also to account for the significant nonlinear elasticity in the exoskeleton – nonlinearity observable in the data and fit work loop (Fig. 3). Further application to the full dataset of Wold et al. (2023) would lead to further insight into the suitability of rate-independent damping as a model of structural dissipation within the insect flight motor.

### Time-domain identification based on transient exoskeletal responses

A second challenge, extending that covered in the previous subsection, is posed by Dudek and Full (2007): the identification of rate-independent damping based on the free response of an insect limb – here, a metathoracic limb of the false death's head cockroach, *Blaberus discoidalis* – following a perturbation. This involves identification of a complete oscillator model, involving rate-independent damping, elasticity and mass, from the perturbation response. The approach of Dudek and Full (2007) to this identification is to assume an oscillator configuration, involving an ideal rate-independent damper; perform a suite of approximate forward oscillator simulations, ignoring the noncausal signal; and then identifying model parameters by matching the forward simulation results. However, there is an alternative inverse approach that is more efficient and more generalisable. Extracting the free displacement response data, *x*(*t*), we can compute a library of oscillator component force terms: elasticity, *x*(*t*); ideal rate-independent damping, 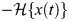; and inertia, 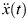. For a linear oscillator:
(18)

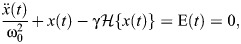
where we have two unknown parameters – natural frequency ω_0_, and dimensionless damping γ – and a residual error E(*t*) that should be zero for an exact fit. We subject E(*t*) to least-squares minimisation and identify ω_0_ and γ directly, an identification not based on forward simulation of the oscillator model, but on its internal consistency.

**Fig. 4.**
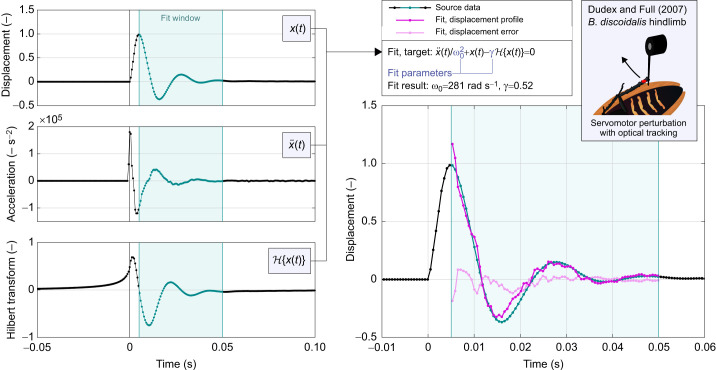
**Time-domain identification of a linear oscillator with ideal rate-independent damping in the perturbation data of** **Dudek and Full (2007)****, for a hindlimb of *Blaberus discoidalis*, via an inverse approach.** Illustrated are the component time signals *x*(*t*) and the derived 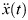 and 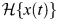, as well as the fit results E_fit_(*t*) and *x*_fit_(*t*). Note that, as per the data in Dudek and Full (2007), displacement is in normalised units. We select a sample window starting after the displacement extrema. This results in a good fit, matching Dudek and Full (2007), whereas fitting prior to this point is poor, either because the servomotor is still in contact with the limb, the acceleration signal data are too coarse, and/or the damper and linear stiffness model breaks down.

 Fig. 4 illustrates this process for a sample free response profile, that reported by Dudek and Full (2007) in their fig. 2B. As an illustration of goodness-of-fit, we may observe the fitted residual E_fit_(*t*), which has the same units as displacement, and may also define a fitted displacement profile as:
(19)

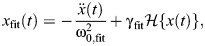

illustrated in Fig. 4. I identify γ=0.52, matching but slightly lower that the estimate of Dudek and Full (2007) for the same profile (γ=0.58). This approach is an illustrative solution to the challenge of full oscillator identification from kinematic data: it is (i) is faster and more accurate than attempting to simulate a causal (or non-causal) oscillator in the time domain, as it requires no forward simulation and thereby completely avoids numerical dissipation; and (ii) can easily account for arbitrary nonlinearities that are hard to simulate, e.g. a polynomial elasticity, which can be added directly to Eqn 18 with no change in identification process. Further application to a wider dataset would again lead to further insight into the suitability of rate-independent damping as a model of insect limb energy dissipation.

### Simulation of exoskeletal structures forward in time

The final challenge that is partly solved by these time-domain methods is that raised across Dudek and Full (2007), Lynch et al. (2022) and Gau et al. (2023): the challenge of simulating general insect exoskeletal models forward in time. In case of the insect flight motor, forward simulations of integrative motor under asynchronous muscle forcing have helped reveal deep properties of these motors, including their evolutionary history (Gau et al., 2023), but structural damping (e.g. as identified in Fig. 3) has been a missing component. A unique feature of the causal damping models is that they are compatible with structural models of the exoskeleton based on initial value problems in time. The causality of these models allows them to be simulated forward in time, with no knowledge of the future, as is typical for structural models, and as is representative of real exoskeletal behaviour.

**Fig. 5.**
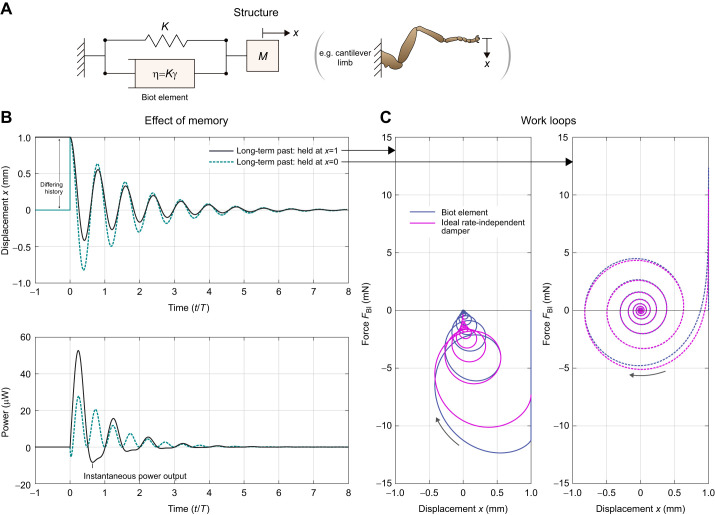
**Forward simulation of the Biot element within a spring-mass-damper system, e.g. representative of a cantilever limb.** (A) Free-body diagram: we consider γ=0.15 and initial conditions *x*=1 and 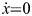. (B) Free response of the spring-mass-damper system. The strong memory of the Biot element has a significant effect: the long-term history of the system, before the initial condition, determines its response, and may lead to power output, i.e. the damper instantaneously doing work. (C) Work loops for the Biot element to both long-term histories, against the work loop for the ideal rate-independent damper (the Hilbert transform) under the same kinematics. The Biot element is a good approximation of the ideal rate-independent damper. However, both dampers output (rather than dissipate) power over a certain time window, representing complex internal energetic behaviour.

Here, I briefly outline simulation of a simple exoskeletal model with structural damping as an example. The model in question (Fig. 5A) is unforced and has linear mass *M*, stiffness *K* and a Biot element with coefficient η=*K*γ, where γ=0.15 is roughly representative of lightly damped exoskeletal structures (Gau et al., 2023; Wold et al., 2023). The model may be expressed as:
(20)

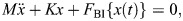

with natural frequency 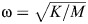, equivalent to natural period 
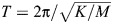
. Discretising Eqn 20, with time vector **t** (elements *t_k_*, constant timestep Δ*t*) and displacement vector **x** (elements *x_k_*), we approximate the second derivative with a first-order backward difference in order to obtain a low-order explicit method for simulation. This yields:
(21)

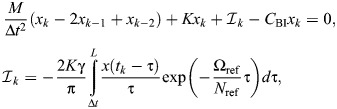
where 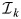 denotes the computation of this ordinary integral by whatever quadrature scheme is chosen, and I use the analytical approximation for *C*_BI_ from the numerical methods described in the Appendix. Note that 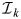 depends only on variables from the previous timesteps (*k*–1, …), as *C*_BI_ accounts for any behaviour at *k*. Then the timestep can be advanced by computing *x_k_* as:
(22)

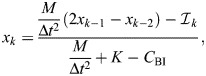
providing a simulation method for this causal system. Fig. 5B,C illustrates the response of this model to an initial condition of *x*=1 mm and 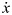
=0 mm s^−1^, e.g. a long perturbation that is then suddenly released.

Two features illustrate that these simulation results are a good representation of causal rate-independent behaviour. First, simulating the model at different natural frequencies (with equivalent tuned Ω_ref_) yields free response profiles that are visually identical to those of Fig. 5B when scaled in time by *T*. This indicates that the damping within the model is frequency independent. Second, the damper forcing, 
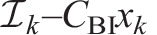
–*C*_BI_*x_k_*, is a good approximation of the forcing of the ideal rate-independent damper (Fig. 5C). This indicates that the scale and waveform of forcing are representative of ideal rate independence. However, note that the numerical scheme is also subject to numerical dissipation; for greater precision, variational approaches could be devised (see Pons and Cirak, 2023).

These simulations reveal several interesting and concerning properties of the Biot element. Because the force response of the Biot element is an integral over past time, the entire system's response is strongly dependent on its long-term history, i.e. its behaviour before the initial condition. Fig. 5B compares this long-term memory effect for two prescribed long-term histories: the system held at *x*=1 mm over an infinite past, and the damper held at *x*=0 mm and then suddenly brought to *x*=1 mm right before the system is released. These two histories lead to free oscillations with large differences (∼100%) in oscillation amplitude. As discussed later, this presence or absence of memory effects in real exoskeleta is one characteristic that may help filter between differing models of rate-independent damping.

This memory effect can also lead to strange energetic properties. Fig. 5B,C compares the instantaneous power dissipation profiles of the Biot element (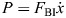) under the two response motions. These power profiles contain regions of power output, or negative dissipation (*P*<0). Here, there are several challenging properties to disentangle. First, in a passive structure, instantaneous power output can still respect conservation of energy provided that this energy is stored and released via a conservative potential, i.e. structural elasticity. The Biot element can be realised as an array of linear dissipative and elastic elements (Fig. 2C), each of which respect conservation of energy, and so the Biot element itself must also respect conservation of energy: instantaneous power output must arise from the elastic elements of the element. However, from the work loops in Fig. 5C, we see that this power output is not consistent with any 1DOF linear or nonlinear parallel or series elasticity, as per Pons and Beatus (2022b, 2023): the Biot element, while conserving energy, behaves as a multiple-DOF rather than 1DOF structure, with internal elements of energy storage. However, by contrast, the ideal rate-independent damper appears to violate conservation of energy directly. This damper has no storage modulus or physical realisation that could provide energy storage and release, and more fundamentally, it is non-causal – without the directionality of time, conservation of energy appears meaningless. The topic of damper energetics leads to the next section.

## ENERGETIC PROPERTIES OF RATE-INDEPENDENT DAMPING

### Non-dissipative behaviour of the ideal rate-independent damper

**Fig. 6.**
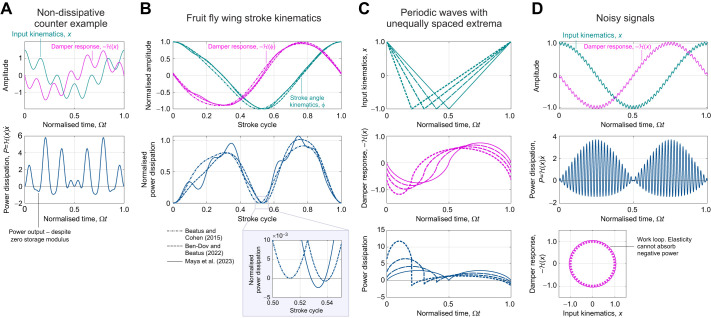
**Energetic properties of the ideal rate-independent damper – a short catalogue of responses to additional waveforms.** (A) The initial non-dissipative counterexample, Eqn 23, in which power output is observed. (B) Wingbeat kinematics from the fruit fly *Drosophila melanogaster*: stroke angle under hovering flight, from several sources (Beatus and Cohen, 2015; Ben-Dov and Beatus, 2022; Maya et al., 2023). Practically, these wingbeat kinematics are dissipative; but strictly, a small level of power output is present (≈0.1% of peak power dissipation). (C) In periodic waves, unequal spacing of the displacement extrema in time can lead to non-dissipative behaviour; the case of a triangle wave is illustrated, partly explaining the power output in (B). The level of power output introduced by this effect is small. (D) The case of noisy signals, illustrated with a deterministic two-harmonic signal, analogous to A. The ideal rate-independent damper produces power when exposed to noise. The damper's work loop under noise excitation illustrates how this power output cannot be explained by any 1DOF linear or nonlinear elasticity (see Pons and Beatus, 2022b, 2023).

In the last subsection, it was shown that both the ideal rate-independent damper and the Biot element were capable of instantaneous power output, for complex energetic reasons. To better understand the implications of this property for exoskeletal modelling, take the ideal-rate independent damper as an illustrative example. This damper has no storage modulus and thus should not store energy. Under simple harmonic motion, it dissipates energy at all times: input *x*
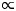
sin*t* yields output *F*∝cos*t* with power dissipation 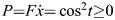. But, to the author's knowledge, it has not previously been observed that these dissipative properties do hold not for general motion: just as all loss moduli are not necessarily causal (Pons, 2024), all loss moduli are not necessarily strictly dissipative. This can be demonstrated by a simple counterexample. Consider motion with just two harmonics:
(23)

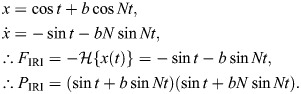
*P*_IRI_ is illustrated for *N*=6 and *b*=0.5 in Fig. 6A. Regions of power output are clearly observed, and as per the previous section, there is a strong case for violation of conservation of energy, though, without deeper theoretical study, we cannot prove rigorously that some type of non-causal energy-conserving structure does not exist. Practically, however, this counterexample highlights that care is needed when treating this damper as purely dissipative, particularly when instantaneous behaviour is under consideration. In lieu of a deeper theoretical characterisation, I provide a catalogue of the damper's behaviour across several key waveforms found in insect biomechanics as follows.

First, the triangle pulse waveform in Figs 2 and 3 is purely dissipative. This applies to other isolated symmetric pulses, e.g. Gaussian, cosine and square pulses, but not to asymmetric versions of these pulses (half-Gaussian, half-cosine, etc.). Conveniently, these properties can be observed directly in tabulated Hilbert transform results (King, 2009); for pure dissipation, the Hilbert transform and pulse velocity always show opposite sign.

Second, dipteran wing stroke angle kinematics, being close to sinusoid, are dissipative for practical purposes. Fig. 6B illustrates this for several recent estimates of the wing stroke kinematics for hovering *Drosophila melanogaster* (Beatus and Cohen, 2015; Ben-Dov and Beatus, 2022; Maya et al., 2023). Strictly, however, these kinematics show a very small level of power output – approximately 0.1% of dissipative peak power.

Third, when a periodic wave has displacement extrema that are unequally spaced in time, non-dissipative behaviour can arise. Fig. 6C illustrates this effect for a triangle wave. In an insect flight motor, this unequal spacing represents, for example, the upstroke being slightly faster than the downstroke, as in *D. melanogaster*, partly explaining the power output described above and in Fig. 6B. However, the power output introduced by this effect is small, and the rate-independent damper may remain a reasonable approximation.

And fourth, noisy motion is not dissipative (Fig. 6D): the addition of high-frequency noise leads to power output in the response, as per the case with multiharmonic components (see Fig. 6A). The effect can be removed by filtering noise out of the signal, but notably this means that rate-independent dampers cannot effectively model a structure's response to noise excitation, e.g. the white noise motion that Wold et al. (2023) applied to hawkmoth thoraces.

### Energetic tuning of the reference frequency in causal dampers

The situation in the causal rate-independent damper and Biot element is more complex, as these structures have a nonzero storage modulus which itself will contribute to instantaneous power output. The construction of these dampers as the limit of generalised Maxwell configurations (see The Biot element and Fig. 2) gives the physical insight that this instantaneous power output arises from the combined action of different energy storage elements (stiffnesses) within the structure, which interact to output energy in a way that cannot be replicated by a single stiffness. To control this behaviour, the reference frequency, Ω_ref_, is available as a free parameter. The role of Ω_ref_ can be described in two equivalent ways. First, Ω_ref_ tunes the damper to have zero storage modulus under sinusoidal motion at certain frequency, equating to pure power dissipation at this frequency. Second, Ω_ref_ defines a frequency threshold: above it, the damper storage modulus acts as positive stiffness; and below it, as negative stiffness (the latter may be unwanted behaviour). The question of whether Ω_ref_ is a real physical property, or just a threshold to exclude unwanted behaviour, is a challenging one (see the Discussion, Origins of rate-independent damping).

**Fig. 7.**
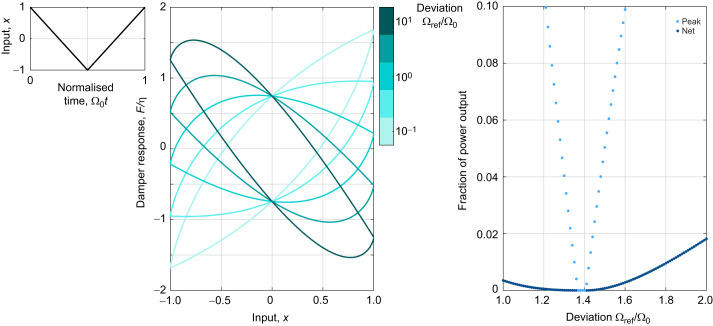
**Energetic tuning of the causal rate-independent damper.** The effect of selecting Ω_ref_≠Ω_0_ for a triangle wave at fundamental frequency Ω_0_ is shown. The work loops of the damper's response under Ω_ref_ illustrates that the power output generated by Ω_ref_ can be completely accounted for by elasticity: a midline elasticity exists, as per Pons and Beatus (2022b, 2023). However, Ω_ref_=Ω_0_ does not lead to no power output; instead, this state is reached at Ω_ref_≈1.39Ω_0_. Away from this point, the level of power output – measured as peak fraction and net (absolute integral) fraction – increases.

Broadly, however, the same classes of energetic behaviour identified in the previous subsection also apply to these dampers, because this behaviour arises from the loss modulus rather than the storage modulus. However, there is an additional energetic effect that is worth isolating. For sinusoidal motion at some frequency Ω, pure dissipation occurs at Ω_ref_=Ω, a transparent energetic tuning relationship. However, for non-sinusoidal periodic waves at fundamental frequency Ω, the value of Ω_ref_ that exactly ensures pure dissipation (if any such value exists) may not be equal to Ω. Fig. 7 illustrates this phenomenon for the response of a causal rate-independent damper to a symmetric triangle wave. Denoting Ω_0_ as the fundamental frequency of this triangle wave, a state of pure dissipation is reached at Ω_ref_≈1.39Ω_0_. If we were to select Ω_ref_=Ω_0_ regardless, power output would be present, to the level of ≈26% of the peak power dissipation and ≈0.35% of the overall absolute work. Several points on this effect should be noted.

Firstly, a general heuristic, following Fig. 7, is as follows: if the waveform is more like a triangle wave than a sinusoid, then the perfect tuning for Ω_ref_ will be higher than Ω_0_, but not likely more than 40% higher (as this is the limit case for a pure triangle wave).

Secondly, for insect wingbeat kinematics under hovering flight, this effect is likely insignificant. For example, *D. melanogaster* wingbeat kinematics tend slightly toward a triangle wave (see Fig. 6B and Dickinson et al., 1999). However, this tendency is sufficiently small that the approximation Ω_ref_=Ω_0_ is suitable: for the kinematics of Maya et al. (2023), the peak power output under this approximation is only ≈0.1% of peak power dissipation.

Finally, two cases in which this effect may be significant are: (1) if the structure is undergoing a transient perturbation, e.g. the triangle perturbation of Fig. 2, in which case measurements of the perturbation timescale cannot be completely relied upon to determine Ω_ref_; and (2) if the structure nominally undergoes sinusoid motion but then this waveform is altered, e.g. an insect flight motor during an extreme manoeuvre (see Gau et al., 2021). In the latter case, the only reliable option is to identify η and Ω_ref_ simultaneously for the data under consideration.

## DISCUSSION

### Origins of rate-independent damping

That rate-independent damping of some form is present in the insect exoskeleton is borne out by experimental data (Dudek and Full, 2006; Gau et al., 2019; Wold et al., 2023), but the physical mechanisms underlying it remain unclear. Several non-exclusive hypotheses can be made, and these hypotheses align in a revealing way with different models of rate-independent damping presented earlier.

The first hypothesis is that this damping represents structural hysteresis arising from elastoplastic behaviour: a transition between recoverable (elastic) and irrecoverable (plastic) deformation as strain increases (Giorgi and Morro, 2021; Zheng, 2019). In metals, the grain-related mesoscopic mechanisms behind this transition are well studied (Weng, 1983; Zheng, 2019). Harder materials within the exoskeleton may show elastoplastic behaviour: there is evidence for plasticity both of the chitin–polymer matrix of insect cuticle (Hillerton et al., 1982) and in chitin itself (Mir et al., 2008), but details are unclear. Elastoplasticity generates true quasistatic hysteresis: energy dissipation is maintained in the quasistatic limit (Ω→0). In a linear model, this corresponds to a constant loss modulus as Ω→0, which we now see is physically impossible: such a linear model is necessarily non-causal and/or shows infinite negative stiffness. This implication here is that true hysteresis is beyond the reach of linear analysis: it is a nonlinear phenomenon that is impossible to linearise without sacrificing basic physical principles. If the causal rate-independent damper or Biot element are used to approximate this hysteresis, then the parameter Ω_ref_ must be regarded as a threshold to exclude unwanted and inaccurate storage modulus trends in the quasistatic limit.

The second hypothesis is that this damping represents a viscoelastic effect that is largely rate independent but remains fundamentally dynamic rather than hysteretic. Here, the Biot element offers the insight that rate-independent damping can arise from an ensemble of mesoscale viscous dampers and linear elasticities (Fig. 2C), analogous to how weakly rate-dependent (fractional-order) damping can arise from a hierarchy of self-similar mesoscale elements within vertebrate cartilaginous tissues (Guo et al., 2021). In the exoskeleton, there are several possibilities for this mesoscale ensemble, extending from the mesoscopic composition of cuticle to the varying thickness and composition of the sclerites (Barbakadze et al., 2006; Casey et al., 2022). Yet, at its core, this viscoelastic damping remains an essentially dynamic process: it cannot show dissipation under true quasistatic motion, and the loss modulus must tend to zero as Ω→0. In this sense it is fundamentally different to true hysteresis, and can be modelled linearly, as per the construction and properties of the Biot element. Under this hypothesis, the Biot element's region of negative storage modulus (as per Ω_ref_) is not necessarily non-physical. When the Biot element is combined with a sufficiently large linear (or nonlinear) stiffness, this negative storage modulus appears simply as decreased quasistatic stiffness, as appears, for example, in experimental DMA data for insect flight muscle (Viswanathan et al., 2020). In this context, Ω_ref_ might be regarded as a physical model parameter.

Distinguishing between these hypotheses does not appear possible with current data, and both effects may be present in the exoskeleton. Model-identification approaches may be an effective approach: each effect has naturally associated models (nonlinear hysteretic versus linear viscoelastic), and testable predictions are available for each. These are discussed below.

### Alternative models and testable predictions

This study began with the classical frequency-domain exoskeletal damping model: the complex stiffness *i*γ. As time-domain formulations reveal, this model is only realistic under harmonic excitation; outside this narrow condition, it observably violates causality. Studying rate-independent damping under general exoskeletal motion requires alternative models, and in this work, two have already been suggested: the causal rate-independent damper and the Biot element. Future prospects for general models of rate-independent damping are inflected by the two hypotheses for its origin that have just been outlined.

In the direction of models for viscoelastic rate-independent damping, the Biot element is a promising candidate. Fractional-order models (Luo and Ikago, 2021) are an alternative with more pronounced rate scaling (i.e. weaker rate-independence), and are currently used in structural models of insect asynchronous muscle (Swank et al., 2006). Considered as a class, these viscoelastic models have a common characteristic: they show a storage modulus that varies with frequency. This varying storage modulus is a key testable prediction of viscoelastic modelling, and an understudied feature in existing structural damping studies. If linear viscoelastic damping is present, then a varying storage modulus will be observed, and if this damping is approximately rate independent, then the storage modulus scaling will be approximately logarithmic. Indeed, evidence for varying storage modulus may already be available: the raw time-series force data of Gau et al. (2019) show increasing peak force with frequency, which may indicate an increasing thoracic storage modulus.

In the direction models for general hysteretic rate-independent damping, the overwhelming consensus of our analysis is that linear hysteresis is non-physical: nonlinear modelling is required. Studies of structural hysteresis in other materials, such as rock (Guyer et al., 1995) and rubber (Penas et al., 2022), suggest several directions for nonlinear modelling, including thermodynamics-driven constitutive modelling with relaxation functions, and assemblies of hysteretic mesoscopic elements, or hysterons (Bertotti and Mayergoyz, 2006; Giorgi and Morro, 2021; Guyer et al., 1995). A key testable prediction of these models, and of quasistatic hysteresis generally, is the phenomenon of endpoint memory (Claytor et al., 2009; Vakhnenko et al., 2005), otherwise known as return-point memory (Zirka and Moroz, 1999), or Madelung's second rule (Brokate and Sprekels, 1996; Penas et al., 2022): the material remembers discrete information relating to recent displacement reversals, or endpoints.

### Implications for insect locomotion

The as-yet unresolved distinction between viscoelastic and hysteric forms of rate-independent damping in the exoskeleton has implications for understanding insect locomotion. Two points of relevance stand out. First, in analyses of exoskeletal mechanisms (e.g. the flight motor), energy dissipation and energy storage are phenomena that are typically considered, and modelled, independently (see Gau et al., 2022; Pons and Beatus, 2022a). Our analysis indicates that, in cases of structural damping, these two effects are closely intertwined. For instance, in the viscoelastic scenario (e.g. the Biot element), a rate-independent loss modulus directly implies a storage modulus that logarithmically increases with frequency. This in turn implies that tuning exoskeletal energy storage to particular locomotive behaviours might be more complex than it appears: the effective stiffness of the exoskeleton may itself vary across behaviours at different frequencies, and quasistatic stiffness measurements (see Jankauski, 2020), may not be completely representative of dynamic motion.

The second point of relevance concerns a commonality between viscoelastic and hysteretic rate-independent damping: the presence of structural memory. The Biot element shows continuous memory (*Q*_BI_; Fig. 2A), whereas hysteretic models show discrete (endpoint) memory. Both memory effects would be testable with current exoskeletal DMA techniques (see Guyer et al., 1997 and Wold et al., 2023), and they bring to mind the topics of physical and/or mechanical intelligence (Blickhan et al., 2007; Sitti, 2021): forms of simple intelligence embodied in passive structure. Energetic memory effects could potentially play a significant role in biolocomotive efficiency. They are, for instance, a challenge to carefully constructed frameworks (Pons and Beatus, 2022b, 2023) that seek to identify the effects of insect wingbeat modulation (frequency and asymmetry) on the flight motor. Hypothetically, by using memory effects to output power at points within the stroke cycle that are not accessible to conventional elasticity, an insect flight motor could violate the elastic-bound conditions (Pons and Beatus, 2022b) and perform wingbeat modulations at lower energetic cost than would otherwise be possible. Structural data precise enough to test this hypothesis have not yet been reported in the literature, but these possibilities motivate further study of rate-independent damping.

### Conclusion

In this work, I developed the first complete time-domain models of rate- (or frequency-) independent damping in insect exoskeleta. I began by translating an established frequency-domain exoskeletal damping model, the ***i***γ model, into the time domain, identifying this time-domain form as the Hilbert transform, and revealing it to be strongly non-causal. Although this noncausality is not problematic under approximately sinusoidal motion, it is more serious under isolated perturbations; for these, significant anticipatory force generation occurs. In response, I extended results from seismic analysis to develop causal time-domain models of exoskeletal rate-independent damping, including an extended form of the Biot element. I developed and validated numerical quadrature techniques for the Hadamard finite-part integrals defining these models, allowing computation of responses to general motion, and simulated the damper within broader structural models. These techniques were applied to several problems in insect biomechanics: identifying thoracic damping from non-sinusoidal DMA data in the presence of nonlinear elasticity; identifying complete limb structural parameters from perturbation kinematics; and simulating exoskeletal structural models in time. In doing so, a key caveat of these models was revealed: they are not purely dissipative, but can output power in ways that are inconsistent with a single nonlinear elasticity. Together, these methods provide: (i) rigorous and complete methods to identify and simulate rate-independent damping in various exoskeletal contexts; (ii) testable predictions to distinguish different models of, and mechanisms behind, exoskeletal damping; and (iii) avenues for further improvement in modelling and understanding this understudied property of the insect exoskeleton.

## APPENDIX

### Numerical evaluation

#### The Hilbert transform

**Fig. A1.**
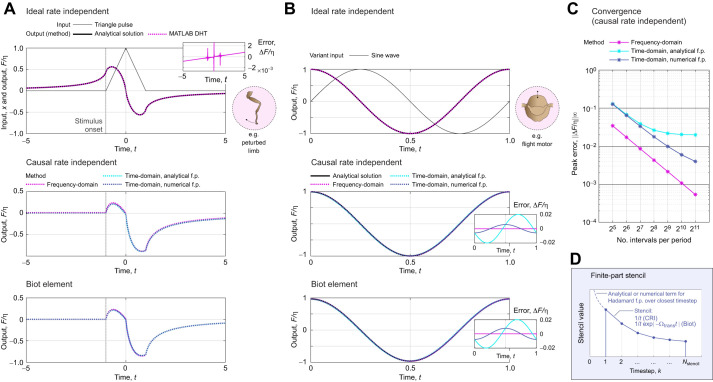
**Performance and illustrative results of different numerical methods for computing the responses of the three rate-independent dampers (ideal, causal and Biot element).** (A) Reponses to a triangle pulse input, representative of a limb perturbation (see Dudek and Full, 2007). Analytical results are available for the ideal damper, via King (2009), permitting an error estimate. (B) Responses to a sine input, representative of flight motor operation (see Pons and Beatus, 2022a). Analytical results are available for all three dampers, enabling an error estimate for the two causal dampers. (C) Convergence of time- and frequency-domain methods for the causal rate-independent damper. Convergence is achieved, though slowly for the time-domain stencil, and this is limited by the (fixed) stencil length. (D) An illustration of the time-domain stencil for computing the finite-part integrals associated with the causal rate-independent damper and Biot element (Eqn A2). Conventional quadrature stops at one timestep away from the present instant, and instead accounts for this closest timestep with a dedicated analytical or numerical approximation (Eqns A3 and A4).

In this short appendix, I provide: (i) a brief description of the numerical methods – some, novel – that were used to evaluate rate-independent damping models under general motion; and (ii) verification that these numerical methods converge to the exact response of these dampers. I begin with the ideal rate-independent damper, which requires evaluation of the Hilbert transform. As mentioned above, existing discrete Hilbert transform (DHT) implementations are widely available: I used hilbert() in MATLAB. Fig. A1 illustrates the performance our implementation for two input motions: a unit triangle pulse (see Fig. 2), for which the exact damper response is (King, 2009):
(rmA1)

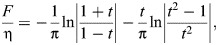

and for a sinusoid, sin2π*t*, for which the exact damper response is *F*/η=cos2π*t*. As can be seen in Fig. A1, the implementation using the DHT accurately captures these responses.

Two further practical points regarding DHT implementation should be noted. First, existing implementations typically output not the Hilbert transform itself but the analytic signal, of which the Hilbert transform is the imaginary part: 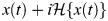 (King, 2009; Marple, 1999). Second, these implementations typically compute the Hilbert transform via the fast Fourier transform (FFT), and thereby assume the input signal to represent a single period of a longer periodic signal. This may be convenient in some contexts (e.g. periodic insect wingbeats), but inconvenient in others (e.g. perturbations of insect limbs). When non-periodic signals are under consideration, they must be zero padded (see Press, 2007).

#### Hadamard finite-part integrals

Computing the finite-part integrals governing the causal rate-independent damper and Biot element is more complex, and requires customised numerical methods. Broadly, two types of method are available: those performed in the frequency domain, via FFT, and those performed directly in the time domain. Both have situational merits.

##### Methods in the frequency domain

Frequency-domain computation of these causal damper responses is convenient; my implementations are illustrated, for validation, in Fig. A1. At some instant *t*, I: (i) FFT the input signal, 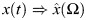; (ii) multiply by the relevant complex modulus to obtain output force, 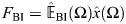 or 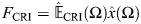; and (iii) inverse FFT, 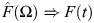. In the case of the causal rate-independent damper, this process contains a weak (logarithmic) singularity in the storage modulus, at 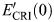. Because this weak singularity is integrable, it suffices to approximate 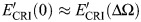, where ΔΩ is the frequency step of the FFT data. Because of the integrability of the singularity, as ΔΩ→0, the damper's response will converge, in the manner of a principal value (p.v.). As a corollary, it is also suitable to perform the computation with an even-number FFT spectrum, not containing Ω=0. In the case of the Biot element, there is no singularity at all. Although this type of evaluation is convenient, its key drawback is that the FFT always takes the input motion, *x*(*t*), to be periodic. If this motion is non-periodic (e.g. Fig. 2), then extensive zero padding is required to prevent bleed over from the assumption of periodicity.

##### Methods in the time domain

Direct time-domain computation of these causal damper responses is also possible via specialised numerical stencils, sets of weights over previous time points. Deriving these stencils requires deeper techniques from the theory of finite-part integrals; these derivations are given in Supplementary Materials and Methods 2, alongside a brief introduction to this theory. Here, only the resulting numerical method is presented, which is illustrated in Fig. A1D. The responses of the causal rate-independent damper and Biot element may computed as:
(rmA2)

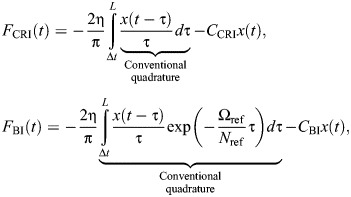
where the annotated term is an ordinary integral that can be approximated by conventional numerical quadrature – I used Simpson's 1/3 rule.

Numerical parameters within Eqn A2 include the current timestep duration (Δ*t*), and the memory length of the damper (*L*), with larger *L* leading to greater precision. *C*_CRI_ and *C*_BI_ are corrector constants that depend on the current timestep duration (Δ*t*) and model parameters (Ω_ref_, *N*_ref_) – they represent the singularity that would otherwise be present in the integral, but has been extracted out (Fig. A1D; Supplementary Materials and Methods 2), Two options for the computation of these corrector constants are available. First are analytical estimates:
(rmA3)

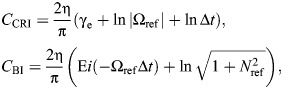
and second are numerical estimates, as the following ordinary integral computations:
(rmA4)

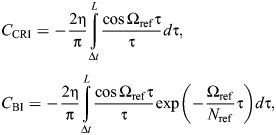


In Eqn A4, it is important that the same quadrature method is used as in Eqn A2, as these correctors can account for certain errors introduced via quadrature. In general, these numerical correctors are preferable: they are more accurate (Fig. A1C) and represent only a small increase in computational cost.

## Supplementary Material

10.1242/jexbio.249940_sup1 Supplementary information
