# Supplementary material for: Causal models of rate-independent damping in insect exoskeleta
Source: J Exp Biol. 2025 Jul 7;228(13):jeb249940. doi: 10.1242/jeb.249940 (PMC12276812; doi:10.1242/jeb.249940)
Supplement: Supplementary information [file jexbio-228-249940-s1.pdf]

Supplementary Materials and Methods

1. Model properties and their derivations

1.1. The causal rate-independent damper

We note here several properties of the causal rate-independent damper.

(**i**) The reference frequency, Ω_{ref}, functions as a linear stiffness parameter, setting the storage modulus to zero at Ω_{ref}, as can be seen via the manipulation:

$$\hat{E}'_{\text{CRI}}(\Omega) = \frac{2}{\pi}\eta \ln\left|\frac{\Omega}{\Omega_{\text{ref}}}\right| = \frac{2}{\pi}\eta \ln|\Omega| - \frac{2}{\pi}\eta \ln|\Omega_{\text{ref}}| \,. \tag{1.1}$$

(**ii**) The time-domain formulation of this causal rate-independent damper can be derived through a memory function (impulse response) approach, though we must pay careful attention to the singularity of the functions involved. Extending the analysis of Makris (1997a) using distributional methods (Estrada and Kanwal, 1989; Graf, 2010; Kanwal, 2004), we may express this memory function as:

$$\begin{aligned} Q_{\text{CRI}}(t) &= \mathcal{F}^{-1}\{\hat{E}'_{\text{CRI}}(\Omega) + i\hat{E}''_{\text{CRI}}(\Omega)\} \\ &= -\frac{\eta}{\pi}\left(\text{p.v.}\frac{1}{t} + \text{f.p.}\frac{1}{|t|} + 2(\gamma_e + \ln|\Omega_{\text{ref}}|)\delta(t)\right) \\ &= -\frac{2\eta}{\pi}\text{f.p.}\frac{\Theta(t)}{t} - \frac{2\eta}{\pi}(\gamma_e + \ln|\Omega_{\text{ref}}|)\delta(t), \end{aligned} \tag{1.2}$$

illustrated in Fig. 2. In Eq. 1.2 we introduce two generalised functions: the Hadamard finite part regularisations (f. p.) of the singular ordinary functions $1/|t|$ and $\Theta(t)/t$, where $\Theta(t)$ is the Heaviside step function, to distributions (Estrada and Kanwal, 1989; Kanwal, 2004; Monegato, 2009). In distributional literature, $\Theta(t)/t$ is often styled t_+^{-1}. The f.p. regularisation in Eq. 14 means that, whenever we integrate or convolve these generalised functions, the integral is defined by Hadamard's finite part: a process for assigning a finite value to diverging (*i.e.*, infinite-valued) integrals by extracting the converging terms in a series expansion of the integral (Jones, 1996). This is necessary because integrals involving $1/|t|$ and $\Theta(t)/t$ often diverge—when so, they cannot be regularised via Cauchy principal value, but require stronger methods[1]. Later in the Supplementary Material (§S2), when we discuss practical numerical methods for evaluating finite part integrals, we provide a brief introduction to their theory.

[1] Hadamard's finite part is not the only option for regularisation, *cf.* Gel'fand and Shilov (1964) and Lighthill (1958). As per Estrada and Kanwal (1989), different regularisations can differ by up to a factor of δ, which be a source of confusion—for instance, seismic analysis literature (Luo and Ikago, 2021; Makris, 1997), following Lighthill (1958), selects regularisations which eliminate the δ term in Eq. 14. Unfortunately, the differing factor of δ is crucial, as it determines an effective stiffness term, $Kx(t)$, in the damper's response (Eq. 15). When we turn to numerical computation in §S2, we will be able to partially resolve this ambiguity via a numerical method for estimating the appropriate δ factor.

Journal of Experimental Biology • Supplementary information

1.2. The Biot element

We note here several properties of the Biot element. For the analysis in this section, and throughout §S2, we define the transition frequency parameter $\Omega_{\mathrm{trans}} = \Omega_{\mathrm{ref}}/\mathrm{N}_{\mathrm{ref}}$ for convenience. This parameter is sometimes styled ε in seismic analysis literature (Luo and Ikago, 2021).

(i) $\widehat{\mathbb{E}}_{\mathrm{BI}}$ is equivalent to the principal branch of the complex logarithm (Ln) (Zill and Shanahan, 2009), providing the concise formulation:

$$\widehat{\mathbb{E}}_{\mathrm{BI}}(\Omega) = \frac{2\eta}{\pi}\mathrm{Ln}\left(1 + i\frac{\Omega}{\Omega_{\mathrm{trans}}}\right) - \frac{2\eta}{\pi}K_{\mathrm{BI}}, \tag{1.3}$$

(ii) Deriving the memory function (impulse response) of the Biot element is a challenging process.To show that Eq. 14 of the main text is the memory function of the Biot element, note that we may express the Fourier transform of the key distribution within $Q_{\mathrm{BI}}(t)$, $\mathrm{f.p.}\,\Theta(t)e^{-\Omega_{\mathrm{trans}}|t|}/t$, via the Fourier convolution theorem as:

$$\mathcal{F}\left\{\mathrm{f.p.}\frac{\Theta(t)}{t}e^{-\Omega_{\mathrm{trans}}|t|}\right\} = \frac{1}{2\pi}\mathcal{F}\left\{\mathrm{p.v.}\frac{e^{-\Omega_{\mathrm{trans}}|t|}}{t}\right\} * \mathcal{F}\{\Theta(t)\} + D, \tag{1.4}$$

where we introduce the indeterminate factor $D\delta$ to account for the fact that switching regularisation methods may cause us to lose a delta distribution in the time domain (Estrada and Kanwal, 1989), *i.e.*, a constant in the frequency domain. We will compute the value of D later. Then, note the established results for the individual Fourier transforms within the convolution on the RHS of Eq. 1.4:

$$\begin{aligned}\mathcal{F}\left\{\mathrm{p.v.}\frac{e^{-\Omega_{\mathrm{trans}}|t|}}{t}\right\} &= -2i\tan^{-1}\left(\frac{\Omega}{\Omega_{\mathrm{trans}}}\right),\\ \mathcal{F}\{\Theta(t)\} &= -i\mathrm{p.v.}\frac{1}{\Omega} + \pi\delta(\Omega),\end{aligned} \tag{1.5}$$

derivable either via hyperfunctions (Graf, 2010) or more classical approaches (Badrieh, 2018; Oberhettinger, 1990). Substituting these terms, the right-hand side of Eq. 1.4 reduces to the computation of a Hilbert transform (the convolution of a function with $\mathrm{p.v.}\,1/\Omega$, *i.e.* $\mathcal{H}$, see Eq. 7 of the main text):

$$\mathcal{F}\left\{\mathrm{f.p.}\frac{\Theta(t)}{t}e^{-\Omega_{\mathrm{trans}}|t|}\right\} = -\mathcal{H}\left\{\tan^{-1}\left(\frac{\Omega}{\Omega_{\mathrm{trans}}}\right)\right\} - i\tan^{-1}\left(\frac{\Omega}{\Omega_{\mathrm{trans}}}\right) + D\,. \tag{1.6}$$

Using further established results for the Hilbert transform (King, 2009), this evaluates to:

$$\mathcal{F}\left\{\mathrm{f.p.}\frac{\Theta(t)}{t}e^{-\Omega_{\mathrm{trans}}|t|}\right\} = -\ln\sqrt{1 + \left(\frac{\Omega}{\Omega_{\mathrm{trans}}}\right)^2} - i\tan^{-1}\left(\frac{\Omega}{\Omega_{\mathrm{trans}}}\right) + D. \tag{1.7}$$

Eq. 1.7 is the complex modulus of the Biot element, $\widehat{\mathbb{E}}_{\mathrm{BI}}(\Omega)$ (Eq. 13 of the main text), up to a factor of $-2\eta/\pi$, the term in K_{BI}, and for some term D.

Journal of Experimental Biology • Supplementary information

Computing D is not necessary to make numerical use of the model (see §S2), but the term is useful to have. To compute D we use an *ad hoc* method based on the response of the model at zero frequency ($\Omega = 0$). At $\Omega = 0$:

$$\widehat{\mathbb{E}}_{\mathrm{BI}}(0) = -\frac{2\eta}{\pi} K_{\mathrm{BI}}, \tag{1.8}$$

that is, the element behaves as a linear spring with stiffness $\widehat{\mathbb{E}}_{\mathrm{BI}}(0)$. Then consider a time-domain evaluation of the damper's response to a constant input, $x = 1$. By Eq. 1.7, the memory function of the element is:

$$Q_{\mathrm{BI}}(t) = -\frac{2\eta}{\pi}\,\mathrm{f.p.}\frac{\Theta(t)}{t} e^{-\Omega_{\mathrm{trans}}|t|} - \frac{2\eta}{\pi} K_{\mathrm{BI}}\delta(t) + C\delta(t), \tag{1.9}$$

with unknown factor C. The constant response, E_0, of Eq. 1.9 to $x = 1$ should equal $\widehat{\mathbb{E}}_{\mathrm{BI}}(0)$, and we seek to compute C such that this occurs. E_0 is given by:

$$\begin{aligned} E_0 &= \left(-\frac{2\eta}{\pi}\,\mathrm{f.p.}\frac{\Theta(t)}{t} e^{-\Omega_{\mathrm{trans}}|t|} - \frac{2\eta}{\pi} K_{\mathrm{BI}}\delta(t) + C\delta(t)\right) * 1 \\ &= -\frac{2\eta}{\pi}\mathrm{f.p.}\int_{-\infty}^{\infty} \frac{\Theta(\tau)}{\tau} e^{-\Omega_{\mathrm{trans}}|\tau|}\, d\tau - \frac{2\eta}{\pi} K_{\mathrm{BI}} + C \\ &= -\frac{2\eta}{\pi}\mathrm{f.p.}\int_{0}^{\infty} \frac{e^{-\Omega_{\mathrm{trans}}\tau}}{\tau}\, d\tau - \frac{2\eta}{\pi} K_{\mathrm{BI}} + C. \end{aligned} \tag{1.10}$$

With a change of variables, $\nu = \Omega_{\mathrm{trans}}\tau$ in the finite-part integral, we can identify its value with the Hadamard regularisation of the Gamma function (Γ) (Paycha, 2012), which for consistency We denote $\mathrm{f.p.}\Gamma$ *in lieu* of Paycha's (2012) Γ^{Had}:

$$\begin{aligned} E_0 &= -\frac{2\eta}{\pi}\mathrm{f.p.}\int_{0}^{\infty} \frac{e^{-\nu}}{\nu}\, d\nu - \frac{2\eta}{\pi} K_{\mathrm{BI}} + C. \\ &= -\frac{2\eta}{\pi}\mathrm{f.p.}\Gamma(0) - \frac{2\eta}{\pi} K_{\mathrm{BI}} + C. \end{aligned} \tag{1.11}$$

$\mathrm{f.p.}\Gamma(0) = -\gamma_e$, the Euler-Mascheroni constant. So, for consistency:

$$\begin{aligned} & E_0 = \widehat{\mathbb{E}}_{\mathrm{BI}}(0) \\ & \therefore \frac{2\eta}{\pi}\gamma_e - \frac{2\eta}{\pi} K_{\mathrm{BI}} + C = -\frac{2\eta}{\pi} K_{\mathrm{BI}}\,, \\ & \therefore C = -\frac{2\eta}{\pi}\gamma_e. \end{aligned} \tag{1.12}$$

This constant is identical to that that appears in the causal hysteretic damper (Eq. 11 of the main text). We may express:

Journal of Experimental Biology • Supplementary information

$$Q_{\mathrm{BI}}(t) = -\frac{2\eta}{\pi}\,\mathrm{f.p.}\frac{\Theta(t)}{t}e^{-\Omega_{\mathrm{trans}}|t|} - \frac{2\eta}{\pi}(\gamma_e + K_{\mathrm{BI}})\delta(t), \tag{1.13}$$

as per Eq. 14 of the main text.

2. Time-domain stencils for finite-part integrals

2.1. Overview of finite-part integration

In this section we provide a brief overview of finite-part integration, and a detailed derivation of the time-domain numerical method for computing the finite-part integrals within the causal rate-independent damper and the Biot element. *First*, regarding finite-part integration—here, we follow Estrada and Kanwal (1994, §2.4), Jones (1996) and Kanwal (2004, §4.2); but additional details can be found in Gel'fand and Shilov (1964). The central concept of the Hadamard finite part is that many types of divergent integrals (*i.e.*, nominally of infinite value) can be split into a diverging term and a unique finite converging term. By discarding the diverging term, the 'finite part' is recovered. Note that this finite part integral *no longer* represents the 'area under the curve' in an intuitive sense: for instance, a finite-part integral may be negative-valued when the integrand is strictly positive. Such an integral might appear to be meaningless, but finite-part integrals of this type arise in certain analyses of real physical phenomena. They have arisen here, in the analysis of rate-independent damping models, and so a method to compute them is required.

To define finite part integration quantitively, consider first the general integral:

$$\int_0^L \frac{\phi(\tau)}{\tau}\,d\tau\,, \tag{2.1}$$

where $\phi(\tau)$ is some function that is integrable over $[0, L]$ (*i.e.*, its own absolute integral converges to a finite value). However, because of the factor $1/\tau$, Eq. 2.1 is a diverging integral, and the point of divergence (singularity) is $\tau = 0$. To see this divergence in action, consider the related integral

$$\int_{\epsilon_1}^L \frac{\phi(\tau)}{\tau}\,d\tau\,, \tag{2.2}$$

for some small ϵ_1. We see that Eq. 2.2 has finite value for finite ϵ_1, but this value does not converge as $\epsilon_1 \to 0$; instead, the integral $\to \pm\infty$. To separate the converging and diverging terms within the integral, we perform the following process. We split the integral up by adding another arbitrary reference point ϵ_2: integration is split into the intervals $[\epsilon_1, \epsilon_2]$ and $[\epsilon_2, L]$. We then perform what appears as a mathematical trick, by adding the term $\phi(0) - \phi(0) = 0$, yielding:

$$\begin{aligned}\int_{\epsilon_1}^L \frac{\phi(\tau)}{\tau}\,d\tau &= \int_{\epsilon_1}^{\epsilon_2} \frac{\phi(\tau)}{\tau}\,d\tau + \int_{\epsilon_2}^L \frac{\phi(\tau)}{\tau}\,d\tau \\ &= \int_0^{\epsilon_2} \frac{\phi(\tau) + \phi(0) - \phi(0)}{\tau}\,d\tau + \int_{\epsilon_2}^L \frac{\phi(\tau)}{\tau}\,d\tau\end{aligned} \tag{2.3}$$

$$= \int_{\epsilon_1}^{\epsilon_2} \frac{\phi(0)}{\tau} d\tau + \int_{\epsilon_1}^{\epsilon_2} \frac{\phi(\tau)-\phi(0)}{\tau} d\tau + \int_{\epsilon_2}^{L} \frac{\phi(\tau)}{\tau} d\tau$$

$$= \underbrace{\phi(0)\ln\epsilon_2}_{A} - \underbrace{\phi(0)\ln\epsilon_1}_{B} + \underbrace{\int_{\epsilon_1}^{\epsilon_2} \frac{\phi(\tau)-\phi(0)}{\tau} d\tau}_{C} + \underbrace{\int_{\epsilon_2}^{L} \frac{\phi(\tau)}{\tau} d\tau}_{D},$$

Now let $\epsilon_2 \to 0$. The terms A, C and D converge when taking this limit (Estrada and Kanwal, 1994). Term B diverges: to define a finite value for the integral in Eq. 2.3, we thus discard this term. This allows us to definite the finite part integral as:

$$\text{f.p.} \int_0^L \frac{\phi(\tau)}{\tau} d\tau = \phi(0)\ln\epsilon + \int_0^{\epsilon} \frac{\phi(\tau)-\phi(0)}{\tau} d\tau + \int_{\epsilon}^{L} \frac{\phi(\tau)}{\tau} d\tau \qquad (2.4)$$

where we now denote $\epsilon = \epsilon_2$. Crucially, this ϵ was a free parameter, and we can confirm (Estrada and Kanwal, 1989; 1994) that Eq. 2.4 gives the same (finite!) result independent of ϵ—we are free to choose, based on numerical or analytical convenience. It is thus typical in this distributional literature to choose $\epsilon = 1$, because then $\ln\epsilon = 0$ and Eq. 2.4 simplifies.

2.2. The causal rate-independent damper: numerical method with analytical corrector

Eq. 2.4 readily yields a basic numerical method for computing the responses of the causal rate-independent damper in the time domain. The time-domain formulation of this damper's response is:

$$\begin{aligned} F_{\text{CRI}}(t) &= -\frac{2\eta}{\pi}\text{f.p.}\int_{-\infty}^{t} \frac{x(\tau)}{t-\tau} d\tau - \frac{2\eta}{\pi}(\gamma_e + \ln|\Omega_{\text{ref}}|)x(t), \\ &= -\frac{2\eta}{\pi}\text{f.p.}\int_0^{\infty} \frac{x(t-\tau)}{\tau} d\tau - \frac{2\eta}{\pi}(\gamma_e + \ln|\Omega_{\text{ref}}|)x(t), \end{aligned} \qquad (2.5)$$

within which is the finite-part integral:

$$\text{f.p.}\int_0^{\infty} \frac{x(t-\tau)}{\tau} d\tau . \qquad (2.6)$$

At this point, to be specific, we restrict ourselves to cases where $x(t-\tau)$ is a function of compact support, *cf.* Kanwal (1997) and Nussenzveig (1972), *i.e.*, input data of finite length. This condition that always be satisfied by physical time-series input data. In this case we can approximate:

$$\text{f.p.}\int_0^{\infty} \frac{x(t-\tau)}{\tau} d\tau \cong \text{f.p.}\int_0^{L} \frac{x(t-\tau)}{\tau} d\tau , \qquad (2.7)$$

for some L, and we know that for sufficiently large L this approximation will be exact. With that formality established, we can identify Eq. 2.7 with Eq. 2.4 under $\phi(\tau) = x(t-\tau)$, and so:

$$\begin{aligned} &\text{f.p.}\int_0^{L} \frac{x(t-\tau)}{\tau} d\tau \\ &= x(t)\ln\epsilon + \int_0^{\epsilon} \frac{x(t-\tau)-x(t)}{\tau} d\tau + \int_{\epsilon}^{L} \frac{x(t-\tau)}{\tau} d\tau . \end{aligned} \qquad (2.8)$$

Journal of Experimental Biology • Supplementary information

Then consider the nature of the function $x(t-\tau)$. For numerical input data, this function is not itself continuous but discrete: its values are given at discrete points (*e.g.*, defined in an array $\mathbf{x}$, with elements x_i, over some discrete time array $\boldsymbol{\tau}$). Here we assume that $\boldsymbol{\tau}$ takes the form $\boldsymbol{\tau} = [0, \Delta\tau, \dots, L]$, *i.e.*, only the first timestep $\Delta\tau$ is relevant. Over this first interval, $[0, \Delta\tau]$, we make the zeroth-order approximation, that $x(\tau) = x(0)$, that is, the continuous value is constant over this timestep. If we make this approximation in Eq. 2.8, and take $\epsilon = \Delta\tau$, then the second term vanishes and we have:

$$\text{f.p.}\int_0^L \frac{x(t-\tau)}{\tau}d\tau \cong x(t)\ln\Delta t + \int_{\Delta t}^L \frac{x(t-\tau)}{\tau}d\tau\,. \tag{2.9}$$

The remaining integral in Eq. 2.9, over $[\Delta\tau, L]$ is an ordinary integral that can be approximated by any relevant quadrature method (trapezoidal, Simpson's, Gaussian). In this article's implementation, Simpson's 1/3 rule is used, with a corrector for the case of an even number of intervals. Returning to the force response of the damper, we have the numerical approximation:

$$\begin{aligned} F_{\text{CRI}}(t) &= -\frac{2\eta}{\pi}\text{f.p.}\int_0^\infty \frac{x(t-\tau)}{\tau}d\tau - \frac{2\eta}{\pi}(\gamma_e + \ln|\Omega_{\text{ref}}|)x(t), \\ &\cong -\frac{2\eta}{\pi}\underbrace{\int_{\Delta t}^L \frac{x(t-\tau)}{\tau}d\tau}_{\substack{\text{conventional}\\ \text{quadrature}}} - \frac{2\eta}{\pi}(\gamma_e + \ln|\Omega_{\text{ref}}| + \ln\Delta t)x(t), \end{aligned} \tag{2.10}$$

2.3. The Biot element: numerical method with analytical corrector

We can analyse the Biot element along similar lines. Noting again the definition of the transition frequency parameter as $\Omega_{\text{trans}} = \Omega_{\text{ref}}/N_{\text{ref}}$, the time-domain formulation of the Biot element's response is:

$$\begin{aligned} F_{\text{BI}}(t) &= -\frac{2\eta}{\pi}\text{ f.p.}\int_{-\infty}^t \frac{x(\tau)}{t-\tau}e^{-\Omega_{\text{trans}}|\tau-t|}d\tau - \frac{2\eta}{\pi}(\gamma_e + K_{\text{BI}})x(t), \\ &= -\frac{2\eta}{\pi}\text{ f.p.}\int_0^\infty \frac{x(t-\tau)}{\tau}e^{-\Omega_{\text{trans}}\tau}d\tau - \frac{2\eta}{\pi}(\gamma_e + K_{\text{BI}})x(t), \end{aligned} \tag{2.11}$$

within which is the finite-part integral:

$$\text{f.p.}\int_0^\infty \frac{x(t-\tau)}{\tau}e^{-\Omega_{\text{trans}}\tau}d\tau \cong \text{f.p.}\int_0^L \frac{x(t-\tau)}{\tau}e^{-\Omega_{\text{trans}}\tau}d\tau\,. \tag{2.12}$$

Considering again discrete x ($\mathbf{x}$) over time array $\boldsymbol{\tau} = [0, \Delta\tau, \dots, L]$, we make the same zeroth-order approximation, $x(\tau) = x(0)$, and so can split Eq. 2.12 directly into:

$$\begin{aligned} &\text{f.p.}\int_0^L \frac{x(t-\tau)}{\tau}e^{-\Omega_{\text{trans}}\tau}d\tau \\ &\quad = x(t)\text{ f.p.}\int_0^{\Delta\tau} \frac{e^{-\Omega_{\text{trans}}\tau}}{\tau}d\tau + \int_{\Delta\tau}^L \frac{x(t-\tau)}{\tau}e^{-\Omega_{\text{trans}}\tau}d\tau\,, \end{aligned} \tag{2.13}$$

where the second integral on the RHS is now again an ordinary integral. We can rearrange the remaining finite-part integral into an expression involving the exponential integral, the special function styled Ei(·):

Journal of Experimental Biology • Supplementary information

$$\begin{aligned} \mathrm{f.p.}\int_0^{\Delta\tau} \frac{e^{-\Omega_{\mathrm{trans}}\tau}}{\tau}\,d\tau &= \mathrm{f.p.}\int_0^{\Omega_{\mathrm{trans}}\Delta\tau} \frac{e^{-\nu}}{\nu}\,d\nu\,, \\ &= \mathrm{f.p.}\int_0^{\infty} \frac{e^{-\nu}}{\nu}\,d\nu - \mathrm{p.v.}\int_{\Omega_{\mathrm{trans}}\Delta\tau}^{\infty} \frac{e^{-\nu}}{\nu}\,d\nu\,, \\ &= \mathrm{f.p.}\,\mathrm{Ei}(0) + \mathrm{Ei}(-\Omega_{\mathrm{trans}}\Delta\tau)\,, \end{aligned} \tag{2.14}$$

where

$$\mathrm{Ei}(y) = -\int_{-y}^{\infty} \frac{e^{-\nu}}{\nu}\,d\nu\,. \tag{2.15}$$

and $\mathrm{Ei}(0)$ must be defined by finite part. From Paycha (2012), $\mathrm{f.p.}\,\mathrm{Ei}(0) = -\gamma_e$. As $\mathrm{Ei}(\Omega_{\mathrm{trans}}\Delta\tau)$ can be evaluated using standard methods (MATLAB function `expint`), we now have a complete numerical method for computing the Biot element's response. We have the finite-part evaluation:

$$\begin{aligned} &\mathrm{f.p.}\int_0^{L} \frac{x(t-\tau)}{\tau} e^{-\Omega_{\mathrm{trans}}\tau}\,d\tau \\ &\quad = (-\gamma_e + \mathrm{Ei}(-\Omega_{\mathrm{trans}}\Delta\tau))x(t) + \int_{\Delta\tau}^{L} \frac{x(t-\tau)}{\tau} e^{-\Omega_{\mathrm{trans}}\tau}\,d\tau\,, \end{aligned} \tag{2.16}$$

which, when substituted into Eq. 2.12 and 2.11, leads to cancellation of the γ_e term, and thus:

$$\begin{aligned} F_{\mathrm{BI}}(t) &= -\frac{2\eta}{\pi}\,\mathrm{f.p.}\int_0^{\infty} \frac{x(t-\tau)}{\tau} e^{-\Omega_{\mathrm{trans}}\tau}\,d\tau - \frac{2\eta}{\pi}(\gamma_e + K_{\mathrm{BI}})x(t), \\ &\cong -\underbrace{\frac{2\eta}{\pi}\int_{\Delta t}^{L} \frac{x(t-\tau)}{\tau} e^{-\Omega_{\mathrm{trans}}\tau}\,d\tau}_{\text{conventional quadrature}} - \frac{2\eta}{\pi}(K_{\mathrm{BI}} + \mathrm{Ei}(-\Omega_{\mathrm{trans}}\Delta t))x(t), \end{aligned} \tag{2.17}$$

2.4. Numerical correctors for the finite part

In Eq. 2.10 and 2.17, the coefficients of $x(t)$, *i.e.*, the factors of $\gamma_e + \ln|\Omega_{\mathrm{ref}}| + \ln \Delta t$ and $K_{\mathrm{BI}} + \mathrm{Ei}(-\Omega_{\mathrm{trans}}\Delta t)$ are effective stiffnesses that correct the ordinary integral term into the relevant finite-part response, tuned to the appropriate reference frequency (Ω_{ref}). As we have seen, significant analytical effort is required to derive these corrector terms. However, we observe that there is an alternate method to compute them, which both validates the analytical correctors and provides an accurate and general technique to potentially replace them.

Considering the causal rate-independent damper as example, we observe the analytical results, Eq. 2.10, indicate that the ordinary integral over $[\Delta t, L]$ can be corrected to the true finite part over $[0, L]$ via some effective stiffness which we term C_{CRI}:

$$F_{\mathrm{CRI}}(t) \cong -\frac{2\eta}{\pi}\int_{\Delta t}^{L} \frac{x(t-\tau)}{\tau}\,d\tau - C_{\mathrm{CRI}}x(t). \tag{2.18}$$

A defining feature of this correction, and thus C_{CRI}, is that the storage modulus of the damper at the frequency Ω_{ref} should be zero. We can estimate this storage modulus directly using Eq. 2.18 by testing the response to a sinusoid $x(t) = \cos(\Omega_{\mathrm{ref}}t)$ and observing the force at $t = 0$:

Journal of Experimental Biology • Supplementary information

$$E'_{\mathrm{CRI}}(\Omega_{\mathrm{ref}}) \cong -\frac{2\eta}{\pi}\int_{\Delta t}^{L}\frac{\cos(-\Omega_{\mathrm{ref}}\tau)}{\tau}d\tau - C_{\mathrm{CRI}}. \quad (2.19)$$

Then, solving for $E'_{\mathrm{CRI}}(\Omega_{\mathrm{ref}}) = 0$, we obtain:

$$C_{\mathrm{CRI}} \cong -\frac{2\eta}{\pi}\int_{\Delta t}^{L}\frac{\cos(\Omega_{\mathrm{ref}}\tau)}{\tau}d\tau\,, \quad (2.20)$$

which is a numerical estimate for C_{BI} which can (and should) be performed by the same quadrature method as used to compute the ordinary damper response (Eq. 2.10). The reason for selecting the same quadrature is that, if there are any errors in the quadrature that specifically lead to an effective stiffness term, this method will eliminate them.

An identical principle can be applied to the Biot element, with only the integral kernel changing. We have:

$$F_{\mathrm{BI}}(t) \cong -\frac{2\eta}{\pi}\int_{\Delta t}^{L}\frac{x(t-\tau)}{\tau}d\tau - C_{\mathrm{BI}}x(t), \quad (2.21)$$

with:

$$C_{\mathrm{BI}} \cong -\frac{2\eta}{\pi}\int_{\Delta t}^{L}\frac{\cos(\Omega_{\mathrm{ref}}\tau)}{\tau}e^{-\Omega_{\mathrm{trans}}\tau}d\tau\,, \quad (2.22)$$

and the relationship between Ω_{ref} and Ω_{trans} being determined by the selected N_{ref}. These two numerical correctors, Eq. 2.20 and Eq. 2.22, are Eq. A3-A4 of the appendix in the main text.

Dataset 1.

Available for download at
https://journals.biologists.com/jeb/article-lookup/doi/10.1242/jeb.249940#supplementary-data

References

Badrieh, F. (2018). *Spectral, Convolution and Numerical Techniques in Circuit Theory*. Berlin, Germany: Springer.

Estrada, R. and Kanwal, R. P. (1989). Regularization, pseudofunction, and hadamard finite part. *J. Math. Anal. Appl.* **141**, 195–207. doi:10.1016/0022-247X(89)90216-3.

Estrada, R. and Kanwal, R. P. (1994). *Asymptotic Analysis*. Basel, Switzerland: Birkhäuser.

Gel'fand, I. M. and Shilov, G. E. (1964). *Properties and Operations*. New York, NY: Academic Press.

Graf, U. (2010). *Introduction to Hyperfunctions and Their Integral Transforms*. Basel, Switzerland: Birkhäuser.

Jones, D. S. (1996). Hadamard's Finite Part. *Math. Methods Appl. Sci.* **19**, 1017–1052. doi:10.1002/(SICI)1099-1476(19960910)19:13<1017::AID-MMA723>3.0.CO;2-2.

Kanwal, R. P. (1997). *Generalized Functions: Theory and Technique*. Basel, Switzerland: Birkhäuser.

King, F. W. (2009). *Hilbert Transforms*. Cambridge, UK: Cambridge University Press.

Lighthill, M. J. (1958). *An Introduction to Fourier Analysis and Generalised Functions*. Cambridge, UK: Cambridge University Press.

Luo, H. and Ikago, K. (2021). Unifying causal model of rate-independent linear damping for effectively reducing seismic response in low-frequency structures. *Earthq. Eng. Struct. Dyn.* **50**, 2355–2378. doi:10.1002/eqe.3450.

Makris, N. (1997). Causal Hysteretic Element. *J. Eng. Mech.* **123**, 1209–1214. doi:10.1061/(ASCE)0733-9399(1997)123:11(1209).

Monegato, G. (2009). Definitions, properties and applications of finite-part integrals. *J. Comput. Appl. Math.* **229**, 425–439. doi:10.1016/j.cam.2008.04.006.

Nussenzveig, H. M. (1972). *Causality and dispersion relations*. New York, NY: Academic Press.

Oberhettinger, F. (1990). *Tables of Fourier Transforms and Fourier Transforms of Distributions*. Berlin, Germany: Springer.

Paycha, S. (2012). *Regularised integrals, sums, and traces: an analytic point of view*. Providence, RI: American Mathematical Society.

Zill, D. G. and Shanahan, P. D. (2009). *A first course in complex analysis with applications*. Sudbury, MA: Jones and Bartlett Publishers.
